# Graves’ disease in children less than 8 years of age: review of clinical features and treatment outcome

**DOI:** 10.1186/1687-9856-2015-S1-P102

**Published:** 2015-04-28

**Authors:** Sophy Korula, Shubha Srinivasan, Geoffrey Ambler, Martin Silink, Neville Howard, Chris Cowell, Paul Benitez-Aguirre, Maria Craig, Kim Donaghue

**Affiliations:** 1 Institute of Diabetes and Endocrinology, The Children’s Hospital, Westmead, Sydney, Australia

## Background

Graves’ disease is the most common cause of thyrotoxicosis in children. Prepubertal children are the most difficult to treat with remission attained in less than 15% [1,2].

Objective: To characterise clinical features and review treatment outcome among children with very early onset Graves’.

## Methodology

Retrospective medical record review of patients diagnosed with Graves’ disease at age less than 8 years, who received treatment in our department in the last 14 years.

## Results

Sixteen patients (2 males) were identified with median age at diagnosis of 4.96 years (range 2.5-7.83). Presenting symptoms were hyperactivity, weight loss, poor sleep and diarrhoea. They had predominant non-Anglo-Saxon ethnicity. Significant co-morbidities were- Down syndrome [1], juvenile idiopathic arthritis [1], situs inversus with extrahepatic biliary atresia ie EHBA [1].Two had family history of Graves’ disease. All had goitre, increased serum Free T4 (median 53.65 pmol/l, range 35-94), increased serum Free T3 (median 33.5 pmol/l, range 19.3-46) and suppressed TSH levels. All were positive for TSII (thyroid stimulating immunoglobulin) or TRAb (thyrotropin receptor antibody). Anti thyroid peroxidise was positive in 83.3% (10/12) and anti thyroglobulin in 80% (8/10). Anti-thyroid drugs (ATD) alone were used in 9 patients, 4 received one dose each of radio-active iodine ablation (10-15 mCi) and 3 underwent thyroidectomy. Our cohort tolerated the ATD’s well- Only 1 had significant liver enzyme elevation (underlying EHBA) after Neomercazole, minor side-effects were: skin rash (3) and arthralgia [1]. Data from patients with more than 30 months follow-up was used to assess outcome. Twelve children with median follow up of 66 months (range: 34-161) and median age of 10.13 years (range: 7.17-16) at last clinic visit qualified. Remission was attained in 58.3% (7/12) - 3 were post thyroidectomy, 1 post radio-active iodine ablation and 3 received only ATD’s. Growth monitoring showed decline in median weight sds from 0.41 at diagnosis to 0.29 at follow up and height sds from 1.35 to 0.69. Of the sixteen patients 2 girls were followed through their puberty till 16 years of age and both are in remission (1 underwent thyroidectomy and other received Neomercazole).

## Conclusion

Our cohort had 16 patients diagnosed with Graves’ at a median age of 4.96 years. Overall remission for those with more than 30 months follow-up is 58.3% (7/12), at a median age of 10.13 years. Thyroidectomy had a remission rate of 100%, ATD’s alone of 33.3% and one dose of radioactive iodine ablation of 25%.
